# Depressive symptoms and co-dependency in caregiver of patients with chronic heart failure

**DOI:** 10.1192/j.eurpsy.2023.1099

**Published:** 2023-07-19

**Authors:** A. Yıldırım, S. Karaca

**Affiliations:** 1 Department of Psychiatric Nursing, Koç University; 2 Department of Psychiatric Nursing, Marmara University, İstanbul, Türkiye

## Abstract

**Introduction:**

Chronic heart failure causes serious mental problems in the life of the patient and caregiver due to symptoms and repeated hospitalizations.

**Objectives:**

This study was conducted to investigate the levels of depression and interdependence in caregivers of patients with chronic heart failure and to examine the relationship of the patient’s depression level with caregiver depression and co-dependence scores.

**Methods:**

The sample of the research, which is descriptive and relationship seeking, consists of 219 volunteer patients with chronic heart failure and caregivers who meet the research criteria. The data were collected using Personal Information Form, Beck Depression Scale and Co-Dependency Assessment Tool, and were evaluated with descriptive statistical analyzes, Kolmogorow-Smirnov, student-t, oneway ANOVA, Pearson correlation analysis and Mann Whitney U Test.

**Results:**

The average age of caregivers was 47.36 ± 12.46 and 60.3% were women. The average age of the patients is 60.70 ± 16.30 and 57.1% are male. Depression was found in 85.8% of patients according to the Beck depression scale score. The presence of depression in the patient and the total depression score of the caregiver (p <0.001), total co-dependency score (p <0.001), self-value (p = 0.001), medical problem (p <0.001) and self neglect (p = 0.005) subscale scores were higher than those who did not have depression. Co-dependence and depression scores are related in caregivers (r=0.367).

**Conclusions:**

There was a positive and significant correlation between the depression levels of the patients and caregivers and the codependence levels of the caregivers, and according to the presence of depression, the mean scores of co-dependence in the caregiver differ.

**Disclosure of Interest:**

None Declared

